# Retraction: Piceatannol inhibits pyroptosis and suppresses oxLDL-induced lipid storage in macrophages by regulating miR-200a/Nrf2/GSDMD axis

**DOI:** 10.1042/BSR-2020-1366_RET

**Published:** 2021-08-05

**Keywords:** atherosclerosis, miR-200a, nrf2, piceatannol, pyroptosis

This article is being retracted from *Bioscience Reports* at the request of the authors. The authors have been unable to replicate the results showing the beneficial effect of piceatannol on oxLDL-induced lipid storage in macrophages, and wish to retract the article. The Editor-in-Chief and Editorial Board agree with the Retraction.

